# Digital back-feeding and the mental health of rural older adults: mediation of basic psychological need satisfaction

**DOI:** 10.3389/fpsyg.2025.1634854

**Published:** 2025-09-12

**Authors:** Qijiao Yang, Haomiao Li, Shengxian Bi, Yingchun Chen

**Affiliations:** ^1^School of Medicine and Health Management, Tongji Medical College, Huazhong University of Science and Technology, Wuhan, Hubei, China; ^2^School of Political Science and Public Administration, Wuhan University, Wuhan, Hubei, China; ^3^Research Centre for Rural Health Service, Key Research Institute of Humanities and Social Sciences of Hubei Provincial Department of Education, Wuhan, China

**Keywords:** digital back-feeding, rural older adults, mental health, basic psychological need satisfaction, China

## Abstract

**Background:**

This study aims to explore the mediating role of basic psychological need satisfaction in the relationship between digital back-feeding and mental health (specifically depression and loneliness) in rural older adults. The objective is to examine how different forms of digital back-feeding influence mental health by enhancing the satisfaction of basic psychological needs.

**Methods:**

This study employed a multistage stratified sampling method, in which 1,663 valid survey responses were collected online. Depression and loneliness in older adults were measured using the Center for Epidemiologic Studies-Depression Scale and UCLA Loneliness Scale-6, respectively. Sociodemographic characteristics were controlled for in the analysis.

**Results:**

Digital access, skills, and literacy were identified as significant negative predictors of depression and loneliness among older adults in rural areas, with basic psychological need satisfaction serving as a key mediator in this relationship. The adverse effects of digital access on depression and loneliness were especially pronounced among men, residents of lower-GDP regions, and individuals from low-income groups.

**Conclusion:**

This study explored the impact of digital back-feeding on the psychological health of older adults in rural areas, with a focus on the mediating role of basic psychological need satisfaction. The results revealed considerable heterogeneity in the effects of digital back-feeding on mental health, with variations across gender, regional economic development, and income levels. Based on these findings, we recommend that policymakers promoting digital empowerment consider these factors to design more targeted and effective intervention strategies.

## 1 Introduction

Against the backdrop of rapid population aging and ongoing digital transformation, older adults living in rural areas in China are facing the dual challenges of mental health problems and the digital divide. Epidemiological studies have consistently shown that the prevalence of depressive and anxiety symptoms among rural seniors is significantly higher than that observed among their urban counterparts (Vander Weele et al., 2012; Du et al., 2019). Such a disparity arises from multiple factors. Geographically, rural areas have limited access to professional mental health services, while the persistent stigma surrounding mental illness further discourages help-seeking among older adults. Moreover, the large-scale migration of younger and middle-aged laborers has weakened traditional family support networks, leaving many rural seniors without timely emotional or practical assistance (Yuan et al., 2020).

At the same time, although digital technologies hold considerable potential for promoting healthy aging (Yu et al., 2024), the urban–rural digital divide places rural older adults at a significant disadvantage in terms of access, usage, and benefits (Akca et al., 2007). Exclusion from digital resources not only reinforces social isolation but also exacerbates existing health inequalities (Hu et al., 2024). Therefore, identifying effective strategies to strengthen both digital inclusion and psychological well-being among rural seniors has become a pressing issue of practical relevance and theoretical importance.

Within this context, digital back-feeding has emerged as a distinctive form of intergenerational support within families, thereby providing a novel approach to addressing the challenges faced by rural older adults (Li and Zhang, 2024). Furthermore, this concept represents a digital-era extension of the traditional cultural back-feeding phenomenon (Zhou and Ding, 2020). At its core, digital back-feeding refers to the process by which younger family members—typically children or grandchildren—actively impart digital skills, knowledge, values, and risk awareness to older generations. In Western scholarship, such intergenerational transmission of technological skills is often described as “bottom-up technological change” (Correa, 2014). However, digital back-feeding encompasses more than just technical instruction—it also represents a form of intergenerational interaction and emotional exchange embedded in everyday practices.

Theoretically, digital back-feeding finds its roots in Margaret Mead’s concept of “postfigurative culture” (Klineberg, 1973), which has since been further developed in the Chinese context under the framework of cultural back-feeding, particularly by scholars such as Zhou. Within the microfamily sphere, digital back-feeding has become the most prevalent and efficient form of support, facilitated by natural emotional bonds and frequent daily interactions. For rural older adults in China, children often serve as the primary source of assistance in navigating digital challenges. On one level, they help their parents overcome initial technological barriers through proxy use (Correa, 2014). On another level, the children enhance older adults’ confidence and self-efficacy. In doing so, digital back-feeding not only promotes digital competence among rural seniors but also contributes to their mental health and overall well-being (Wang and Wu, 2022; Luo et al., 2024).

To further explore the mechanisms through which digital back-feeding influences mental health, this study employs self-determination theory (SDT) as the primary theoretical framework. In accordance with SDT, the satisfaction of basic psychological needs constitutes a critical pathway for promoting overall well-being. These needs encompass three core dimensions: autonomy (the sense that one’s actions are self-initiated and self-endorsed), competence (the feeling of being capable of effectively navigating and mastering one’s environment), and relatedness (the experience of maintaining warm, supportive relationships and a sense of belonging) (Deci and Ryan, 2000). The present study hypothesizes that digital back-feeding may exert positive effects on mental health by fulfilling these fundamental psychological needs. Prior research suggests that digital back-feeding can enhance older adults’ social participation and foster a sense of digital empowerment (Neves and Amaro, 2012). Related to this, SDT provides a robust theoretical framework to explain these outcomes.

Although the positive effects of digital back-feeding have been preliminarily explored, several limitations remain. First, most existing studies have concentrated on urban populations, paying limited attention to rural older adults—a group particularly vulnerable to digital exclusion. Second, empirical research directly linking digital back-feeding to mental health outcomes remains scarce, and the psychological mechanisms underlying this relationship have yet to be thoroughly investigated. To address these gaps, we focus on rural older adults in China and introduce the mediating role of basic psychological need satisfaction into the analysis of the relationship between digital back-feeding and mental health. By doing so, we aim to enrich the existing literature and provide a stronger theoretical foundation for designing targeted interventions and informing policy development.

In light of these considerations, the present study aims to examine the relationship between digital back-feeding and mental health outcomes—specifically depression and loneliness—among rural older adults in China, employing a mediation model emphasizing the role of basic psychological need satisfaction. The findings are expected to provide empirical evidence to support the development of family-based digital back-feeding interventions and to inform effective strategies for enhancing digital literacy and psychological well-being among rural seniors.

## 2 Literature review

### 2.1 Digital back-feeding and mental health

Digital back-feeding refers to the process whereby younger generations leverage their digital advantages to teach older generations how to access, use, and understand digital devices, online services, and related applications (Bell, 1968; Suter and Norwood, 2017). This concept comprises three dimensions—access, skills, and literacy—each addressing different aspects of the intergenerational digital divide. Access back-feeding focuses on providing support for hardware, software, and connectivity; skills back-feeding involves the transfer of operational knowledge and practical usage abilities (Lee and Kim, 2019); and literacy back-feeding extends to shaping digital cognition, risk awareness, and lifestyle concepts, thereby representing a deeper form of cultural influence. This three-dimensional framework is employed in existing research to examine the mechanisms and social significance of digital back-feeding. At the level of mental health, digital back-feeding enhances older adults’ psychological well-being by fostering parent–child interactions and greater emotional support (Sampasa-Kanyinga et al., 2020). In this bidirectional educational process, parents and children—through role reversal, dialogue, and collaboration—not only facilitate the transfer of digital skills but also strengthen intergenerational emotional resonance and mutual understanding, thereby narrowing generational gaps and reducing conflicts (Eynon and Helsper, 2015). As the primary setting for digital back-feeding, the family plays a vital role in providing intergenerational support within older adults’ broader social support networks. Empirical evidence further shows that emotional and instrumental support from children can substantially alleviate older adults’ loneliness and social isolation, ultimately mitigating negative emotions, such as depression, among this population (Ren and Klausen, 2024).

Despite its benefits, digital back-feeding carries potential risks, particularly in varying cultural contexts and implementation models where these risks can be amplified. For example, in rural areas, a proxy-based approach is often employed, which may help older adults gain short-term access to digital society. However, by fostering dependency and diminishing autonomy, this can hinder the long-term development of digital literacy (Reifegerste et al., 2020). Therefore, an ideal digital back-feeding model should combine “teaching” and “proxy” strategies, which offer support while encouraging active engagement and exploration by older adults.

Furthermore, cultural specificity plays a crucial role. In Chinese families, influenced by Confucian filial piety and traditional intergenerational feedback models, there is a heightened emphasis on children’s caregiving responsibilities and emotional bonds. Here, digital back-feeding takes on the ethical dimensions of “digital filial piety,” which is characterized by significant emotional investment and sustained continuity (Pan et al., 2022; Chou, 2011). In contrast, Western family dynamics tend to prioritize individual autonomy, with technical support being more sporadic, pragmatic, and less emotionally charged. Consequently, in China, digital back-feeding transcends mere skills transfer to become an emotional practice of intergenerational care and identity reconstruction (Correa, 2014). Existing research suggests that emotional support from children to older adults plays a more pivotal role in improving mental and physical health than purely informational or technical support (Lang and Schütze, 2002). If digital back-feeding can strike a balance between technical empowerment and emotional support, it holds greater promise in reducing depression, alleviating loneliness, and enhancing the overall psychological well-being of older adults. Based on the above analysis, this study proposes the following hypothesis:

*H1*: Digital back-feeding (including access, skills, and literacy back-feeding) has a positive promoting effect on the mental health of rural senior adults in China.

### 2.2 Digital back-feeding and basic psychological need satisfaction

Digital back-feeding not only enhances intergenerational interaction and emotional support but also contributes to the fulfillment of older adults’ basic psychological needs across multiple dimensions (Thalhammer and Schmidt-Hertha, 2015). Grounded in SDT, three primary mechanisms can be identified. First, digital back-feeding provides a unique context for intergenerational interaction in which patient instruction and emotional feedback from adult children strengthen feelings of belonging and familial connectedness, thereby satisfying the older adults’ need for relatedness (Kalmijn and Saraceno, 2008). Second, through the gradual acquisition of digital skills, older adults develop technological competence and the ability to cope with digital challenges. As such, this process enhances their self-efficacy and fulfills the need for competence (Eynon and Helsper, 2015). Third, digital back-feeding enables older adults to more effectively utilize digital tools to access information, participate in social activities (Hargittai et al., 2019), and manage daily life independently, thus reinforcing perceived control and fulfilling the need for autonomy.

Within the Chinese familial and cultural context, digital back-feeding is deeply embedded in the ethical framework of filial piety and intergenerational responsibility. This practice emphasizes patience, respect, and reciprocal communication, thus fostering a supportive environment characterized by trust and frequent interaction. These conditions are especially conducive to the fulfillment of psychological needs. In contrast, digital support in Western societies tends to adopt a more functional orientation, with comparatively limited emphasis on emotional dimensions, which may restrict its psychological benefits. Building on this reasoning, the following hypothesis is proposed:

*H2*: Digital back-feeding positively influences the satisfaction of basic psychological needs among older adults in rural China.

### 2.3 Basic psychological need satisfaction and mental health

SDT posits that individuals possess three innate psychological needs: relatedness, competence, and autonomy. The satisfaction of these needs fosters the development of positive self-concepts and enhances interpersonal relationships, thereby contributing to better psychological functioning. Importantly, while these needs are inherent, their satisfaction is not automatic. Instead, it is an internal process influenced by external environmental factors and individuals’ internal psychological resources (Deci and Ryan, 2000). Thus, the satisfaction of basic psychological needs can serve as a crucial mediating mechanism linking external contexts, such as family dynamics and digital back-feeding, to individual psychological well-being.

Empirical evidence has consistently validated this mechanism across various populations and cultural contexts. For example, studies have shown that basic psychological need satisfaction mediates the relationship between family environmental factors and adolescents’ psychological development (Cao et al., 2022). In the context of older adults in rural China, who are navigating the process of digital inclusion, external support in the form of digital back-feeding can promote the satisfaction of these basic psychological needs. This, in turn, may reduce depressive symptoms and loneliness, while enhancing overall psychological well-being. Based on this reasoning, the following hypothesis is proposed:

*H3*: Basic psychological need satisfaction mediates the relationship between digital back-feeding and psychological health among older adults in rural China.

**Figure 1:**
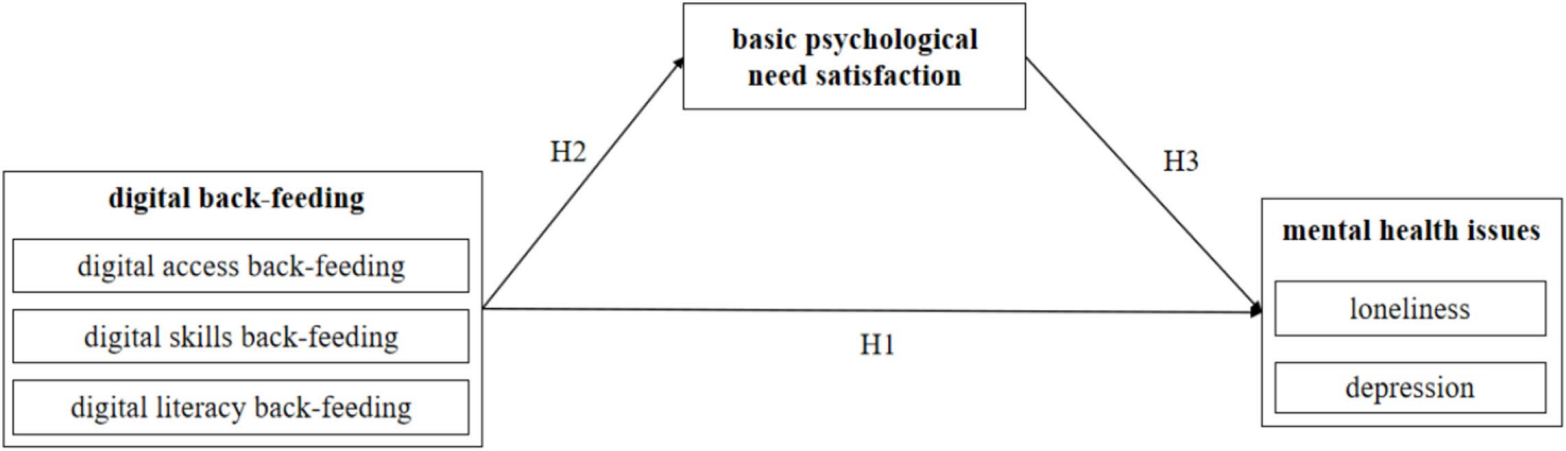
Framework of the study.

The theoretical framework of this study is presented in Figure 1.

## 3 Methods

### 3.1 Data and participants

To ensure representativeness and methodological rigor, this study employed a multistage stratified random sampling approach. Province H, situated in central China, was chosen as the sampling region due to its balanced geographical distribution and moderate level of economic development, making it a representative model for typical rural areas in central China.

The sampling procedure consisted of four stages. First, Province H was identified as the focal province based on its demographic characteristics and socioeconomic comparability to other central provinces. Second, two administrative units—County E and City X—were randomly selected from the province using a simple random sampling method. Third, using a random number table, 10 communities or administrative villages were randomly chosen within each selected county/city. Finally, within each selected community/village, household-based surveys were conducted with older adults who met the following inclusion criteria: (1) aged 60 years or above; (2) resided locally for at least 6 months within the 12 months prior to the survey; and (3) provided informed consent and voluntarily participated. At this final stage, convenience sampling was applied within each community/village. In total, 1,663 valid responses from older adults were collected, yielding a sample size adequate for robust statistical analyses.

### 3.2 Variables

#### 3.2.1 Implicit variable

Psychological health was assessed using two indicators: depression and loneliness. Depression was measured with the Center for Epidemiologic Studies Depression Scale (CES-D). Originally developed by Radloff (1977) and later simplified by Andersen et al. (1994), the 10-item short form CES-D captures affective, behavioral, and somatic dimensions, with items such as “being bothered by trivial things,” “feeling fearful,” and “feeling lonely.” Respondents rated the frequency of symptoms experienced during the past week on a four-point Likert scale (0 = rarely or none of the time, 1 = some of the time, 2 = occasionally, 3 = most of the time). Positive items were reverse-coded, and higher total scores indicated more severe depressive symptomatology. In this study, the mean depression score was 10.11 (SD = 4.95) with good internal consistency (Cronbach’s *α* = 0.874).

Loneliness was assessed with the six-item short form of the UCLA Loneliness Scale (ULS-6), a widely applied tool in gerontological research developed to reduce respondent burden while retaining strong validity (Neto, 2014). The scale demonstrated excellent internal reliability in this study (Cronbach’s α = 0.947) with a mean score of 11.18 (SD = 4.12).

#### 3.2.2 Independent variable

Digital back-feeding. Based on prior research, digital back-feeding was conceptualized across three dimensions: digital access, digital skills, and digital literacy. “Digital access back-feeding” refers to adult children’s assistance in helping older adults obtain digital devices and connectivity services, such as purchasing smartphones, providing internet access, or installing commonly used applications. “Digital skills back-feeding” captures the guidance provided by adult children in teaching older adults how to operate digital devices and applications. “Digital literacy back-feeding” encompasses efforts to enhance older adults’ understanding of digital culture and to provide education on online safety and risk awareness (Lee and Kim, 2019).

Based on prior literature (Correa, 2016; Galperin and Arcidiacono, 2019; Nelissen and Van den Bulck, 2018), a Digital Back-feeding Scale was developed in which all items were measured as dichotomous variables (0 = “never received,” 1 = “received”). Digital access back-feeding was assessed through several items, such as “Do you own a digital device?” and “What is the source of the device?” digital skills back-feeding through questions such as “Do you use WeChat/Douyin (TikTok)?” “Can you perform operations such as mobile payments, QR code scanning, or use of mini-programs?” and “Can you complete online services, such as medical appointment booking, ticket purchasing, or QR-code ordering?”; and digital literacy back-feeding through items, such as “Do you know how to search for and evaluate information content?” “Are you aware of online security precautions?” and “Have you been introduced to popular internet culture, such as emojis or trending expressions?”

#### 3.2.3 Mediating variable

Basic psychological need satisfaction was assessed using the Chinese version of the Basic Psychological Need Satisfaction Scale (BPNS), revised by Deci and Ryan and further adapted by Liu and colleagues (Deci and Ryan, 2000). Grounded in SDT, the scale consists of 19 items across three dimensions: autonomy (6 items, e.g., “I generally feel free to express my own opinions”), competence (6 items, e.g., “People who know me well think I am capable of handling tasks effectively”), and relatedness (7 items, e.g., “I really enjoy the people I interact with”). Responses were rated on a seven-point Likert scale, with nine items reverse-coded. Higher scores indicated greater satisfaction with basic psychological needs. In this study, the mean score was 92.63 (SD = 12.80) with good internal reliability (Cronbach’s *α* = 0.863).

#### 3.2.4 Control variates

To account for potential confounding effects, a series of control variables was included. For sociodemographic characteristics, we included region (classified by GDP level: 1 = lower-GDP regions, 2 = higher-GDP regions), gender (1 = male, 2 = female), age (years), ethnicity (1 = Han, 2 = ethnic minority), education (1 = no schooling, 2 = incomplete primary school, 3 = completed primary school, 4 = junior high school or above), marital status (1 = married, 0 = not married), living arrangement (1 = living alone, 2 = living with spouse and children, 3 = living with children only, 4 = living with spouse only, 5 = other), retirement status (1 = retired, 0 = not retired), and monthly income in the past year (1 = no income, 2 = 1–500 RMB, 3 = 501–1,000 RMB, 4 = 1,001–2000 RMB, 5 = above 2000 RMB). Family support variables included whether adult children provided financial support (1 = yes, 0 = no) and whether the respondents provided care for grandchildren (1 = yes, 0 = no). Additionally, health status was measured by the number of chronic conditions (0 = none, 1 = one, 2 = two or more). The selection of these control variables was informed by prior research (Pinquart and Sörensen, 2001; Koesten, 2004; Nelissen and Van den Bulck, 2018), as these factors may simultaneously influence digital back-feeding and psychological health outcomes.

### 3.3 Statistical analysis

Data were processed and analyzed in this study using Stata 18.0. Initially, descriptive statistics and correlation analyses were conducted. Then, we employed a mediation model based on the bootstrap method (5,000 resamples) to test whether basic psychological need satisfaction mediates the relationship between digital back-feeding and psychological health. All regression models controlled for the aforementioned variables. To ensure the robustness of the results, maximum likelihood estimation was used for parameter estimation.

## 4 Results

### 4.1 Descriptive statistics

As shown in Table 1, among the 1,663 older adults aged 60 and above, 814 received digital access back-feeding (48.95%), 407 received digital skills back-feeding (24.47%), and 905 received digital literacy back-feeding (54.42%).

**Table 1:** Statistical descriptions of variables (*n* = 1,663).

Variable	Mean	SD	Min	p50	Max
Region	1.210	0.408	1	1	2
Sex	1.523	0.500	1	2	2
Age	71.357	6.258	60	71	98
Ethnicity	1.260	0.439	1	1	2
Education	2.345	1.080	1	2	4
Marital status	0.707	0.455	0	1	1
Residency	2.915	1.163	1	3	5
Retired or not	0.444	0.497	0	0	1
Monthly income	2.282	1.088	1	2	5
Whether children are financially supportive	0.521	0.500	0	1	1
Whether they take care of grandchildren	0.115	0.320	0	0	1
Number of chronic conditions	0.877	0.738	0	1	2
Digital access back-feeding	0.489	0.500	0	0	1
Digital skills back-feeding	0.245	0.430	0	0	1
Digital literacy back-feeding	0.544	0.498	0	1	1
Depression	10.111	4.948	3	9	33
Loneliness	11.184	4.125	6	12	24
Basic psychological need satisfaction	92.628	12.802	28	94	132

### 4.2 Correlation analysis

Correlation analysis (Table 2) revealed that digital access back-feeding, digital skills back-feeding, and digital literacy back-feeding were each significantly and positively associated with basic psychological need satisfaction (*r* = 0.08, *p* < 0.001; *r* = 0.18, *p* < 0.001; *r* = 0.14, *p* < 0.001, respectively). In turn, basic psychological need satisfaction was significantly and negatively correlated with depressive symptoms (*r* = −0.68, *p* < 0.001) and loneliness (*r* = −0.50, *p* < 0.001).

**Table 2:** Correlation coefficient for each variable (*n* = 1,663).

Variables	M ± SD	1	2	3	4	5	6
1 depression	10.11 ± 4.95	——					
2 loneliness	11.18 ± 4.13	0.54***	——				
3 digital access back-feeding	0.49 ± 0.50	−0.09***	−0.08**	——			
4 digital skills back-feeding	0.25 ± 0.43	−0.27***	−0.19***	0.36***	——		
5digital literacy back-feeding	0.54 ± 0.50	−0.13***	−0.14***	0.67***	0.42***	——	
6basic psychological need satisfaction	92.63 ± 12.80	−0.68***	−0.50***	0.08***	0.18***	0.14***	——

*p < 0.1, **p < 0.05, ***p < 0.01.

### 4.3 Mediation analysis results

Controlling for relevant variables, further analyses were conducted to examine the impact of digital back-feeding from children on the psychological health of older adults. We estimated each mediation model using maximum likelihood with 5,000 bootstrap replications. As shown in Table 3, the results indicated that basic psychological need satisfaction significantly mediated the relationships between all three dimensions of digital back-feeding and both depression and loneliness.

**Table 3:** Mediating effect analysis of digital back-feeding and mental health.

Path	Effect	Boot SE	95% BootCI	*p*
Digital Access Back-Feeding → Basic Psychological Need Satisfaction → Depression	−0.350	0.015	−0.064 ~ −0.007	<0.001
Digital Access Back-Feeding → Basic Psychological Need Satisfaction → Loneliness	−0.200	0.010	−0.045 ~ −0.005	<0.001
Digital Skills Back-Feeding → Basic Psychological Need Satisfaction → Depression	−0.786	0.013	−0.095 ~ −0.043	<0.001
Digital Skills Back-Feeding → Basic Psychological Need Satisfaction → Loneliness	−0.448	0.010	−0.066 ~ −0.029	<0.001
Digital Literacy Back-Feeding → Basic Psychological Need Satisfaction → Depression	−0.562	0.015	−0.086 ~ −0.027	<0.001
Digital Literacy Back-Feeding → Basic Psychological Need Satisfaction → Loneliness	−0.319	0.010	−0.060 ~ −0.018	<0.001

**Figure 2:**
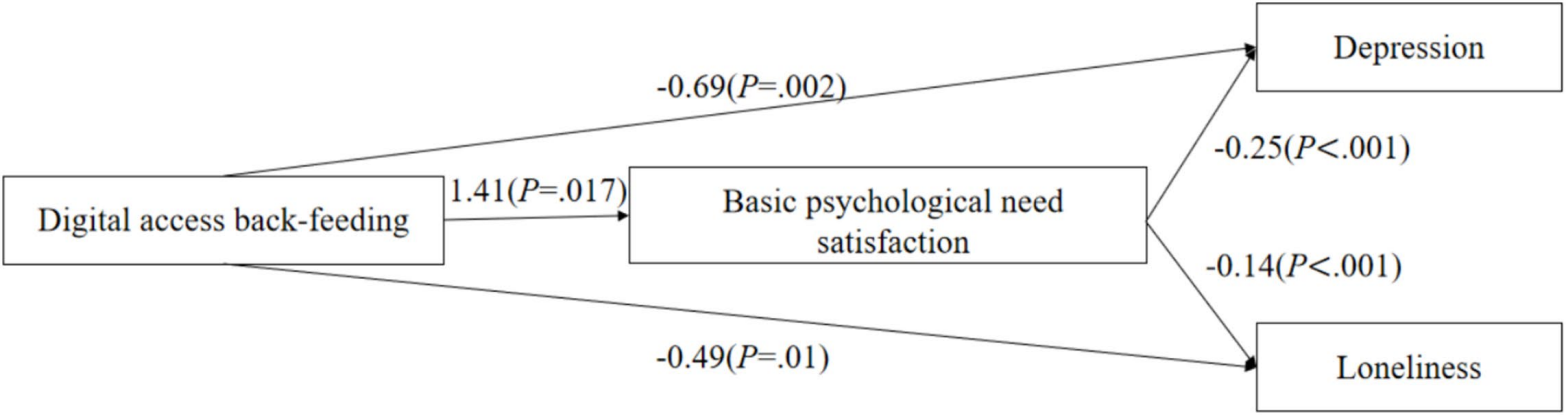
Digital access back-feeding and the mediating effect model of psychological health.

**Figure 3:**
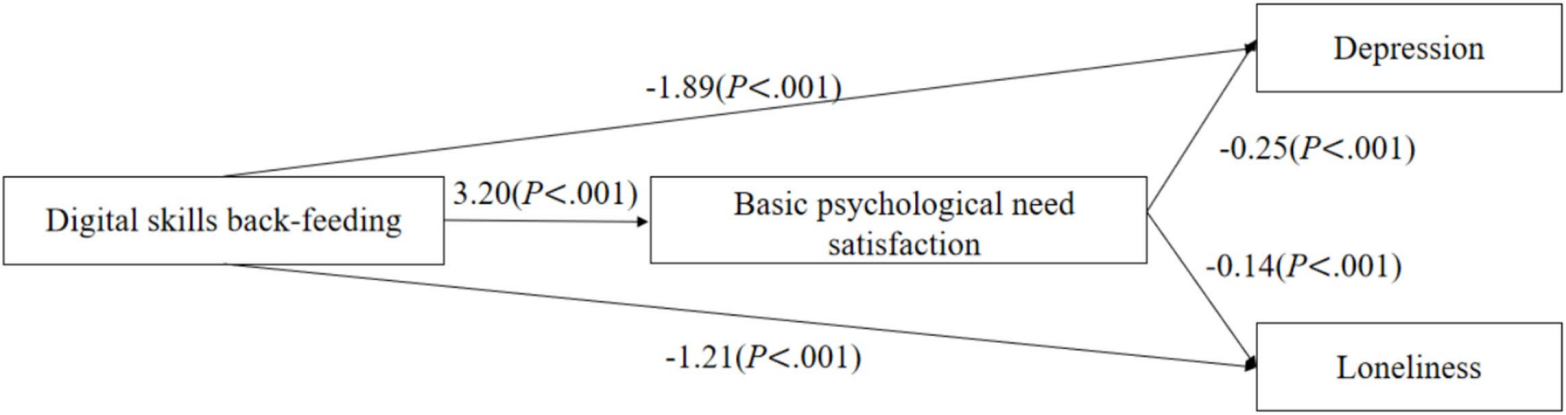
Digital skills back-feeding and the mediating effect model of psychological health.

**Figure 4:**
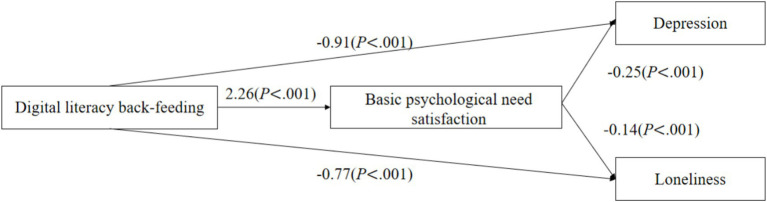
Digital literacy back-feeding and the mediating effect model of psychological health.

As shown in Figures 2–4, digital back-feeding from children was directly associated with improved psychological health among older adults. Specifically, digital access back-feeding was significantly related to fewer depressive symptoms (*β* = −0.69, *p* = 0.002) and lower loneliness (*β* = −0.49, *p* = 0.01). Similarly, digital skills back-feeding exhibited significant negative associations with depressive symptoms (*β* = −1.89, *p* < 0.001) and loneliness (*β* = −1.21, *p* < 0.001). Furthermore, digital literacy back-feeding was significantly associated with reduced depressive symptoms (*β* = −0.91, *p* < 0.001) and lower loneliness (*β* = −0.77, *p* < 0.001).

When basic psychological need satisfaction was incorporated as a mediator in the models, significant indirect effects were observed across all three dimensions of digital back-feeding. Specifically, digital access back-feeding (*β* = 1.41, *p* = 0.017), digital skills back-feeding (*β* = 3.20, *p* < 0.001), and digital literacy back-feeding (*β* = 2.26, *p* < 0.001) all significantly enhanced basic psychological need satisfaction. In turn, higher levels of basic psychological need satisfaction significantly reduced depressive symptoms (*β* = −0.25, *p* < 0.001) and loneliness (*β* = −0.14, *p* < 0.001).

Overall, the results suggest that basic psychological need satisfaction functions as a robust mediator linking the three dimensions of digital back-feeding to the psychological well-being of older adults.

### 4.4 Heterogeneity analysis

#### 4.4.1 Gender

In terms of gender, as shown in Table 4, basic psychological need satisfaction significantly mediated the relationship between digital back-feeding and psychological health among men but not women. For men, significant mediation effects were observed for digital skills back-feeding with loneliness (95% BootCI: −0.072 to −0.013) and depressive symptoms (95% BootCI: −0.108 to −0.021), as well as for digital literacy back-feeding with loneliness (95% BootCI: −0.079 to −0.005) and depressive symptoms (95% BootCI: −0.121 to −0.008). In contrast, no significant mediation was found for digital access back-feeding in relation to either loneliness (95% BootCI: −0.035 to 0.032) or depressive symptoms (95% BootCI: −0.054 to 0.050).

**Table 4:** Mediating effect analysis of digital back-feeding and mental health by gender.

Path	Male			Female		
	Effect	Boot SE	95% BootCI	Effect	Boot SE	95% BootCI
Digital Access Back-Feeding → Basic Psychological Need Satisfaction → Loneliness	−0.011	0.017	−0.035 ~ 0.032	0.095	0.019	−0.023 ~ 0.051
Digital Skills Back-Feeding → Basic Psychological Need Satisfaction → Loneliness	−0.402	0.015	−0.072 ~ −0.013	−0.273	0.015	−0.057 ~ 0.001
Digital Literacy Back-Feeding → Basic Psychological Need Satisfaction → Loneliness	−0.345	0.019	−0.079 ~ −0.005	−0.120	0.020	−0.055 ~ 0.023
Digital Access Back-Feeding → Basic Psychological Need Satisfaction → Depression	−0.019	0.027	−0.054 ~ 0.050	0.168	0.027	−0.035 ~ 0.072
Digital Skills Back-Feeding → Basic Psychological Need Satisfaction → Depression	−0.722	0.022	−0.108 ~ −0.021	−0.482	0.021	−0.080 ~ 0.001
Digital Literacy Back-Feeding → Basic Psychological Need Satisfaction → Depression	−0.619	0.029	−0.121 ~ −0.008	−0.212	0.029	−0.078 ~ 0.033

#### 4.4.2 GDP level

Table 5 presents the results by regional economic context. In lower-GDP regions, basic psychological need satisfaction significantly mediated the relationships between digital skills back-feeding and loneliness (95% BootCI: −0.067 to −0.019) and depressive symptoms (95% BootCI: −0.094 to −0.028), as well as between digital literacy back-feeding and loneliness (95% BootCI: −0.057 to −0.000) and depressive symptoms (95% BootCI: −0.081 to −0.001). However, in higher-GDP regions, no significant mediation was detected, and the indirect effects of digital access back-feeding were nonsignificant in both contexts.

**Table 5:** Mediating effect analysis of digital back-feeding and mental health at different GDP levels.

Path	Lower-GDP regions			Higher-GDP regions		
	Effect	Boot SE	95% BootCI	Effect	Boot SE	95% BootCI
Digital Access Back-Feeding → Basic Psychological Need Satisfaction → Loneliness	0.086	0.014	−0.017 ~ 0.037	−0.209	0.032	−0.094 ~ 0.037
Digital Skills Back-Feeding → Basic Psychological Need Satisfaction → Loneliness	−0.435	0.012	−0.067 ~ −0.019	0.162	0.014	−0.005 ~ 0.049
Digital Literacy Back-Feeding → Basic Psychological Need Satisfaction → Loneliness	−0.234	0.015	−0.057 ~ −0.000	−0.515	0.038	−0.146 ~ 0.003
Digital Access Back-Feeding → Basic Psychological Need Satisfaction → Depression	0.154	0.019	−0.024 ~ 0.053	−0.346	0.071	−0.199 ~ 0.082
Digital Skills Back-Feeding → Basic Psychological Need Satisfaction → Depression	−0.779	0.017	−0.094 ~ −0.028	0.268	0.030	−0.012 ~ 0.105
Digital Literacy Back-Feeding → Basic Psychological Need Satisfaction → Depression	−0.419	0.021	−0.081 ~ −0.001	−0.851	0.075	−0.291 ~ 0.008

#### 4.4.3 Income level

As presented in Table 6, mediation effects were significant among lower-income groups but not among higher-income groups. Specifically, for individuals with no income, basic psychological need satisfaction significantly mediated the associations between digital access back-feeding and loneliness (95% BootCI: −0.089 to −0.005) and depressive symptoms (95% BootCI: −0.160 to −0.018), as well as between digital skills back-feeding and loneliness (95% BootCI: 0.002 to 0.048) and depressive symptoms (95% BootCI: 0.008 to 0.087). In comparison, no significant mediation was detected for digital literacy back-feeding in this group. Among participants with a monthly income ≤500 RMB, basic psychological need satisfaction significantly mediated the effects of digital skills and digital literacy back-feeding on loneliness and depressive symptoms. In contrast, for those with a monthly income above 500 RMB, no significant mediation effects emerged across any of the three dimensions of digital back-feeding.

**Table 6:** Mediating effect analysis of digital back-feeding and mental health at different income levels.

Path	No income			≤ 500 RMB			501–1,000 RMB			1,001–2000 RMB			≥ 2001 RMB		
	Effect	Boot SE	95% BootCI	Effect	Boot SE	95% BootCI	Effect	Boot SE	95% BootCI	Effect	Boot SE	95% BootCI	Effect	Boot SE	95% BootCI
Digital Access Back-Feeding → Basic Psychological Need Satisfaction → Loneliness	−0.271	0.022	−0.089 ~ −0.005	0.241	0.020	−0.010 ~ 0.067	−0.659	0.062	−0.207 ~ 0.043	0.188	0.035	−0.039 ~ 0.100	0.162	0.040	−0.050 ~ 0.107
Digital Skills Back-Feeding → Basic Psychological Need Satisfaction → Loneliness	0.199	0.012	0.002 ~ 0.048	−0.330	0.015	−0.065 ~ −0.004	−0.127	0.052	−0.123 ~ 0.090	−0.464	0.032	−0.122 ~ 0.004	−0.254	0.038	−0.115 ~ 0.040
Digital Literacy Back-Feeding → Basic Psychological Need Satisfaction → Loneliness	−0.030	0.016	−0.038 ~ 0.026	−0.585	0.022	−0.112 ~ −0.027	−0.351	0.070	−0.183 ~ 0.099	−0.078	0.034	−0.078 ~ 0.054	0.454	0.044	−0.012 ~ 0.162
Digital Access Back-Feeding → Basic Psychological Need Satisfaction → Depression	−0.711	0.036	−0.160 ~ −0.018	0.407	0.028	−0.014 ~ 0.096	−0.623	0.055	−0.185 ~ 0.031	0.362	0.053	−0.062 ~ 0.151	0.324	0.063	−0.087 ~ 0.163
Digital Skills Back-Feeding → Basic Psychological Need Satisfaction → Depression	0.522	0.020	0.008 ~ 0.087	−0.556	0.021	−0.091 ~ −0.006	−0.120	0.043	−0.097 ~ 0.075	−0.892	0.047	−0.182 ~ 0.007	−0.510	0.059	−0.164 ~ 0.066
Digital Literacy Back-Feeding → Basic Psychological Need Satisfaction → Depression	−0.078	0.032	−0.074 ~ 0.056	−0.986	0.031	−0.159 ~ −0.038	−0.332	0.058	−0.149 ~ 0.085	−0.149	0.052	−0.118 ~ 0.086	0.910	0.064	−0.021 ~ 0.234

## 5 Discussion

In this study, we explored the mediating role of basic psychological need satisfaction in the relationship between digital back-feeding and the psychological well-being of rural older adults. Consistent with our three hypotheses, the results demonstrated that digital back-feeding had a positive effect on the psychological well-being of rural older adults, that basic psychological need satisfaction was positively associated with psychological well-being, and that it functioned as a significant mediator linking digital back-feeding to psychological well-being, thus supporting H1, H2, and H3, respectively.

First, the results of this study validate the existence of the “digital back-feeding → basic psychological need satisfaction → psychological well-being” pathway, thus providing empirical support for SDT. Specifically, all three forms of digital back-feeding (digital access, digital skills, and digital literacy) significantly enhanced basic psychological need satisfaction (*β* = 1.41, *p* = 0.017 for digital access; *β* = 3.20, *p* < 0.001 for digital skills; and *β* = 2.26, *p* < 0.001 for digital literacy), which, in turn, alleviated depression (*β* = −0.25, *p* < 0.001) and loneliness (*β* = −0.14, *p* < 0.001) among rural older adults. This mechanism suggests that digital back-feeding promotes psychological well-being by fulfilling older adults’ basic psychological needs. In particular, skill- and literacy-based back-feeding not only enhance technological competence but also strengthen self-efficacy and social recognition, thereby contributing to improved psychological outcomes (She et al., 2025). This finding aligns with domestic and international studies, which emphasize that digital empowerment for rural older adults extends beyond technical assistance and serves as a critical safeguard for psychological well-being (Sun and Zhou, 2021; Lei et al., 2023; Liu et al., 2025). However, the marginal effect of digital access back-feeding on psychological well-being is comparatively weaker, suggesting a notable gap between “being connected” and “being able to use and benefit from technology.” This is consistent with existing evidence indicating that digital usage ability has a stronger impact on older adults’ psychological well-being than mere access (Yu et al., 2024; Chen et al., 2023; Yang et al., 2025). Our findings further demonstrate that improvements in digital skills and literacy are more effective than simple access in translating digital back-feeding into positive psychological outcomes.

Second, the heterogeneity analysis highlights the critical role of gender differences in the relationship between digital back-feeding and psychological health. Among men, basic psychological need satisfaction significantly mediated the effects of digital skills and digital literacy back-feeding on loneliness and depressive symptoms, whereas no such effects were observed among women. Specifically, for men, the mediation effects were significant for digital skills back-feeding (on loneliness and depression) and digital literacy back-feeding (on loneliness and depression), while the mediation effects of digital access back-feeding were not significant. From the perspectives of gender role and intergenerational support theories, these findings suggest that, in old age, digital back-feeding in the form of skills and literacy enables men to reconstruct social roles and personal value, thereby fulfilling autonomy and competence needs, as emphasized in SDT, which, in turn, leads to improvements in loneliness and depression (Pinquart and Sörensen, 2001; Kim et al., 2017). In contrast, according to a study, rural women are more likely to rely on traditional family and community-based support networks. Furthermore, their psychological health is not only shaped by digital back-feeding but also by cultural expectations and caregiving responsibilities traditionally associated with women (Jiang et al., 2024). These findings underscore the necessity of incorporating gender sensitivity into digital empowerment policies and tailoring interventions to the specific needs of male and female older adults to more effectively promote psychological well-being.

Third, the heterogeneity analysis by regional GDP level and individual income emphasizes that the mediating effects of digital back-feeding are particularly pronounced in economically disadvantaged contexts and among low-income groups. Specifically, in lower-GDP regions, basic psychological need satisfaction significantly mediated the associations between digital skills back-feeding and both loneliness and depressive symptoms, as well as between digital literacy back-feeding and both outcomes. However, no significant mediation effects were found for digital access back-feeding. This finding underscores the marginal effects of digital back-feeding in resource-constrained environments, that is, in low-income and low-GDP areas, improvements in digital skills not only represent a breakthrough in technology access but also serve as a crucial pathway for fulfilling basic psychological needs (Xiang and Xing, 2025; Cui et al., 2024). In these contexts, older adults—who often face limited opportunities to engage with modern technologies due to economic constraints—derive substantial benefits from their children’s digital back-feeding, which provides them with valuable social resources that significantly improve their psychological well-being. In contrast, in more economically developed regions or among higher-income groups, older adults already have greater access to social support and mental health resources, thereby limiting the marginal benefits of digital back-feeding.

Finally, the mediating role of basic psychological need satisfaction supports the applicability of SDT in the context of digital intergenerational interventions. However, cultural specificities must also be considered. For instance, due to differences in demographic aging and family communication dynamics, China and Western societies follow distinct trajectories in eldercare. In China, family-based care remains the dominant model, driven not only by the willingness of adult children to provide support but also by older adults’ desire for intergenerational intimacy and family harmony. Furthermore, within the Confucian tradition of familialism, the intensity of relatedness need satisfaction far exceeds that typically observed in Western contexts. Thus, given the underdeveloped state of the social care sector and the enduring influence of filial piety, Chinese intergenerational relations are characterized by a reciprocal “support and return” pattern in which children assume caregiving responsibilities for their parents, thereby linking intergenerational exchanges throughout the life course (Chou, 2011). In comparison, Western societies place greater emphasis on individual autonomy. Thus, while Western scholarship often frames digital tutoring by children as a form of “bottom-up technological transfer” (Correa, 2014), the Chinese family context underscores identity reversal and emotional tension within this process. Moreover, while previous studies have affirmed the positive role of children’s ICT support in helping older adults adopt new media, our findings suggest that emotional support from children may be even more critical than purely informational or technical assistance in enhancing older adults’ psychological well-being (Lang and Schütze, 2002).

The abovementioned findings also carry important policy and intervention implications. First, digital empowerment policies should be designed with greater attention to gender differences. Our results indicated that men experienced significant improvements in psychological health through digital skills and literacy back-feeding, whereas similar mediating effects were not observed among women. Accordingly, policy initiatives should prioritize men’s needs in digital empowerment, particularly by enhancing their digital skills and literacy. In comparison, for women, interventions may be more effective if they emphasize broader social support networks, such as family- and community-based mental health programs, rather than relying solely on digital back-feeding as the primary pathway of intervention.

Second, targeted efforts are needed to strengthen digital skills training and mental health support in economically disadvantaged regions. The study results demonstrated that in lower-GDP areas, digital skills and literacy back-feeding significantly improved older adults’ psychological health. Thus, to reduce the urban–rural divide and promote digital equity, governments and social organizations must increase investments in digital education in these regions. Beyond providing basic digital access, programs should focus on enhancing older adults’ digital skills and literacy, complemented by mental health interventions tailored to the needs of these populations to alleviate loneliness and depression.

Finally, special attention should be given to low-income groups. Our findings revealed that basic psychological need satisfaction significantly mediated the link between digital back-feeding and psychological health among low-income individuals. Therefore, governments should strengthen digital empowerment and mental health support for this population, especially those with no or very low monthly income. Specific practical measures may include offering free or low-cost digital training programs, accessible mental health services, and family support initiatives, which can help reduce technological barriers while simultaneously alleviating psychological problems driven by economic stress.

## 6 Limitations and suggested future research directions

Several limitations of this study should be acknowledged. First, while useful for revealing correlations between variables, the cross-sectional design does not capture the dynamic nature of the back-feeding internalization and cannot rule out potential bidirectional causal relationships. Therefore, future research should adopt a longitudinal design to better elucidate the causal relationships among these variables. Additionally, we did not fully address the issue of endogeneity in the mediator variables. Thus, future research could employ various methods, such as instrumental variable analysis or difference-in-differences approaches to mitigate endogeneity bias, thereby enhancing the reliability of causal inferences.

Second, the dependent variables in this study were measured using binary indicators, which may not fully capture the complexity and diversity of digital back-feeding behaviors. To more accurately reflect the multidimensional nature of digital back-feeding, future studies could employ more refined measurement tools, such as continuous scales or multidimensional assessments. Doing so can improve the precision and reliability of the measurements used.

Third, while this study demonstrated the mediating role of basic psychological need satisfaction in the relationship between digital back-feeding and mental health, further exploration of the underlying psychological mechanisms remains limited. As such, future research could delve deeper into the specific psychological processes behind this mediation, using more detailed measurement tools to gain a more comprehensive understanding of how digital back-feeding influences older adults’ mental health through the fulfillment of psychological needs.

Finally, although a multistage stratified random sampling strategy was employed, convenience sampling was applied at the final stage of household recruitment, which may have introduced potential selection bias. Additionally, all variables were measured through self-reported questionnaires, which may be subject to social desirability or recall bias.

## 7 Conclusion

In conclusion, this study demonstrates that digital back-feeding—through access, skills, and literacy—can significantly enhance the mental health of rural older adults, with basic psychological need satisfaction functioning as a central mediating mechanism. The findings highlight important heterogeneities, indicating that men, residents of economically disadvantaged regions, and low-income groups benefit most from digital empowerment. These results underscore the necessity of incorporating gender, regional, and income considerations into digital inclusion strategies. By aligning digital empowerment initiatives with the fulfillment of older adults’ psychological needs, policymakers can not only promote digital equity but also contribute to reducing health disparities and fostering the mental well-being of an aging society.

## Data Availability

The original contributions presented in the study are included in the article/supplementary material, further inquiries can be directed to the corresponding author.
